# Supplementary material for: A single early-in-life macrolide course has lasting effects on murine microbial network topology and immunity
Source: Nat Commun. 2017 Sep 11;8:518. doi: 10.1038/s41467-017-00531-6 (PMC5593929; doi:10.1038/s41467-017-00531-6)
Supplement: Supplementary file 1 — Supplementary Information [file 41467_2017_531_MOESM1_ESM.pdf]

Description of Supplementary Files

File Name: Supplementary Information
Description: Supplementary Figures and Supplementary Tables

File Name: Supplementary Data 1
Description: Significantly expressed genes after one and three tylosin doses, related to Figure 2, Panel a.

File Name: Supplementary Data 2
Description: Ileal immune gene expression in SPF and GF mice treated with PAT, related to Figure 3, Panel g.

File Name: Peer Review File

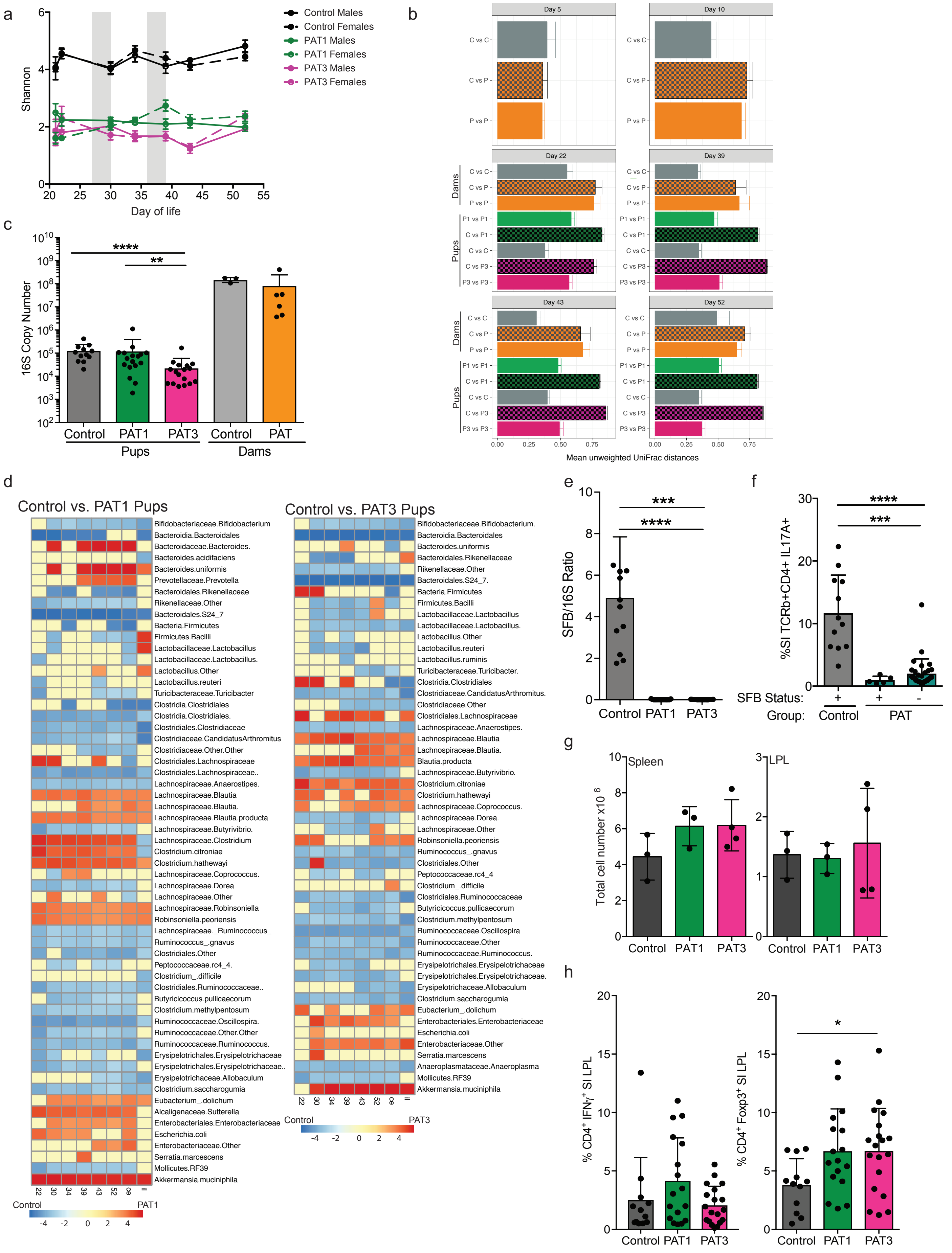
a
Shannon
Day of life
Control Males
Control Females
PAT1 Males
PAT1 Females
PAT3 Males
PAT3 Females
b
Day 5
Day 10
Day 22
Day 39
Day 43
Day 52
C vs C
C vs P
P vs P
P1 vs P1
C vs P1
C vs P3
P3 vs P3
Dams
Pups
Mean unweighted UniFrac distances
c
16S Copy Number
****
**
Control
PAT1
PAT3
PAT
Pups
Dams
d
Control vs. PAT1 Pups
Bifidobacteriaceae.Bifidobacterium
Bacteroidia.Bacteroidales
Bacteroidaceae.Bacteroides.
Bacteroides.acidifaciens
Bacteroides.uniformis
Prevotellaceae.Prevotella
Bacteroidales.Rikenellaceae
Rikenellaceae.Other
Bacteroidales.S24_7
Bacteria.Firmicutes
Firmicutes.Bacilli
Lactobacillaceae.Lactobacillus
Lactobacillaceae.Lactobacillus.
Lactobacillus.Other
Lactobacillus.reuteri
Turicibacteraceae.Turicibacter
Clostridia.Clostridiales
Clostridia.Clostridiales.
Clostridiales.Clostridiaceae
Clostridiaceae.CandidatusArthromitus
Clostridiaceae.Other.Other
Clostridiales.Lachnospiraceae
Clostridiales.Lachnospiraceae..
Lachnospiraceae.Anaerostipes.
Lachnospiraceae.Blautia
Lachnospiraceae.Blautia.
Lachnospiraceae.Blautia.producta
Lachnospiraceae.Butyrivibrio.
Lachnospiraceae.Clostridium
Clostridium.citroniae
Clostridium.hathewayi
Lachnospiraceae.Coprococcus.
Lachnospiraceae.Dorea
Lachnospiraceae.Other
Lachnospiraceae.Robinsoniella
Robinsoniella.peoriensis
Lachnospiraceae._Ruminococcus_
Ruminococcus_.gnavus
Clostridiales.Other
Peptococcaceae.rc4_4.
Clostridium_.difficile
Clostridiales.Ruminococcaceae..
Butyricicoccus.pullicaecorum
Clostridium.methylpentosum
Ruminococcaceae.Oscillospira.
Ruminococcaceae.Other.Other
Ruminococcaceae.Ruminococcus.
Erysipelotrichales.Erysipelotrichaceae
Erysipelotrichales.Erysipelotrichaceae..
Erysipelotrichaceae.Allobaculum
Clostridium.saccharogumia
Eubacterium_.dolichum
Alcaligenaceae.Sutterella
Enterobacteriales.Enterobacteriaceae
Escherichia.coli
Enterobacteriaceae.Other
Serratia.marcescens
Mollicutes.RF39
Akkermansia.muciniphila
Control
PAT1
Control vs. PAT3 Pups
Bifidobacteriaceae.Bifidobacterium.
Bacteroidia.Bacteroidales
Bacteroides.uniformis
Bacteroidales.Rikenellaceae
Rikenellaceae.Other
Bacteroidales.S24_7.
Bacteria.Firmicutes
Firmicutes.Bacilli
Lactobacillaceae.Lactobacillus
Lactobacillaceae.Lactobacillus.
Lactobacillus.Other
Lactobacillus.reuteri
Lactobacillus.ruminis
Turicibacteraceae.Turicibacter.
Clostridia.Clostridiales
Clostridiaceae.CandidatusArthromitus.
Clostridiaceae.Other
Clostridiales.Lachnospiraceae
Lachnospiraceae.Anaerostipes.
Lachnospiraceae.Blautia
Lachnospiraceae.Blautia.
Blautia.producta
Lachnospiraceae.Butyrivibrio.
Clostridium.citroniae
Clostridium.hathewayi
Lachnospiraceae.Coprococcus.
Lachnospiraceae.Dorea.
Lachnospiraceae.Other
Robinsoniella.peoriensis
Ruminococcus_.gnavus
Clostridiales.Other
Peptococcaceae.rc4_4
Clostridium_.difficile
Clostridiales.Ruminococcaceae
Butyricicoccus.pullicaecorum
Clostridium.methylpentosum
Ruminococcaceae.Oscillospira
Ruminococcaceae.Other
Ruminococcaceae.Ruminococcus.
Erysipelotrichales.Erysipelotrichaceae
Erysipelotrichales.Erysipelotrichaceae.
Erysipelotrichaceae.Allobaculum
Clostridium.saccharogumia
Eubacterium_.dolichum
Enterobacteriales.Enterobacteriaceae
Escherichia.coli
Enterobacteriaceae.Other
Serratia.marcescens
Anaeroplasmataceae.Anaeroplasma
Mollicutes.RF39
Akkermansia.muciniphila
Control
PAT3
e
SFB/16S Ratio
***
****
f
%SI TCRb+CD4+ IL17A+
SFB Status:
Group:
g
Spleen
LPL
Total cell number x10 6
h
% CD4+ IFNγ+ SI LPL
% CD4+ Foxp3+ SI LPL
*

Supplementary Figure 1. Microbial community composition after one or three antibiotic courses. (a) Mean (± SEM) unrarified α-diversity over time in control, PAT1 and PAT3 groups using the Shannon evenness metric. Solid lines represent female pups Control (n=3), PAT1 (n=8), PAT3 (n=9) and dashed lines represent male pups Control (n=9), PAT1 (n=9), PAT3 (n=10). Statistical analysis performed using two-sample t-test with Monte Carlo permutations, for statistical significance, see Supplementary Table 1. (b) Comparisons between the mean pairwise unweighted UniFrac intra- and inter-group divergences for antibiotic-exposed dams and pups over the course of the experiment. Intra and intergroup unweighted UniFrac distances were averaged over independently drawn sample pairs (subsampled without replacement and replicated 999 times), (For significance, Supplementary Table 2). (c) Mean (± SD) fecal 16S rRNA DNA copy number in pups and dams at sacrifice, pups at P52; Control (n=12), PAT1 (n=17), and PAT3 (n=19) groups and dams at 20 weeks of age (pup P61); Control (n=3), PAT (n=6). (d) Significantly differential taxa, ($p<0.05$), in control (blue) and PAT1 or PAT3 (red) fecal, cecal (ce), or ileal (il) samples in pups (n=same as panel a) over the course of the experiment, using the Linear discriminant analysis Effect Size (LefSe) statistical tool. Significant taxa were plotted using the aheatmap R package. (e) Mean (± SD) SFB copy numbers in fecal samples at P52 in (n=12), PAT1 (n=17), and PAT3 (n=19) groups, as determined by qPCR, normalized by total 16S rRNA copy number. (f) Mean (± SD) frequency of small intestine lamina propria $CD4^{+}IL17A^{+}$ cells in SFB-positive or -negative (n=12), PAT1 (n=17), and PAT3 (n=19) mice. (g) Mean (± SD) absolute splenic and intestinal lamina propria viable cell counts, as detected by trypan blue exclusion (Control, n=3; PAT1, n=3; PAT3, n=3). (h) Mean (± SD) frequency of small intestine lamina propria $CD4^{+}IFN\gamma^{+}$ cells and $CD4^{+}$ $Foxp3^{+}$ cells in control (n=12), PAT1 (n=17), PAT3 (n=19). For panels c, e, f, g and h, significance testing performed using one-way analysis of variance with Tukey post-hoc test or Kruskal-Wallis non-parametric test with Dunnett's multiple comparisons test. For all panels: *$p<0.01$, **$p<0.01$, ***$p<0.001$, ****$p<0.0001$.

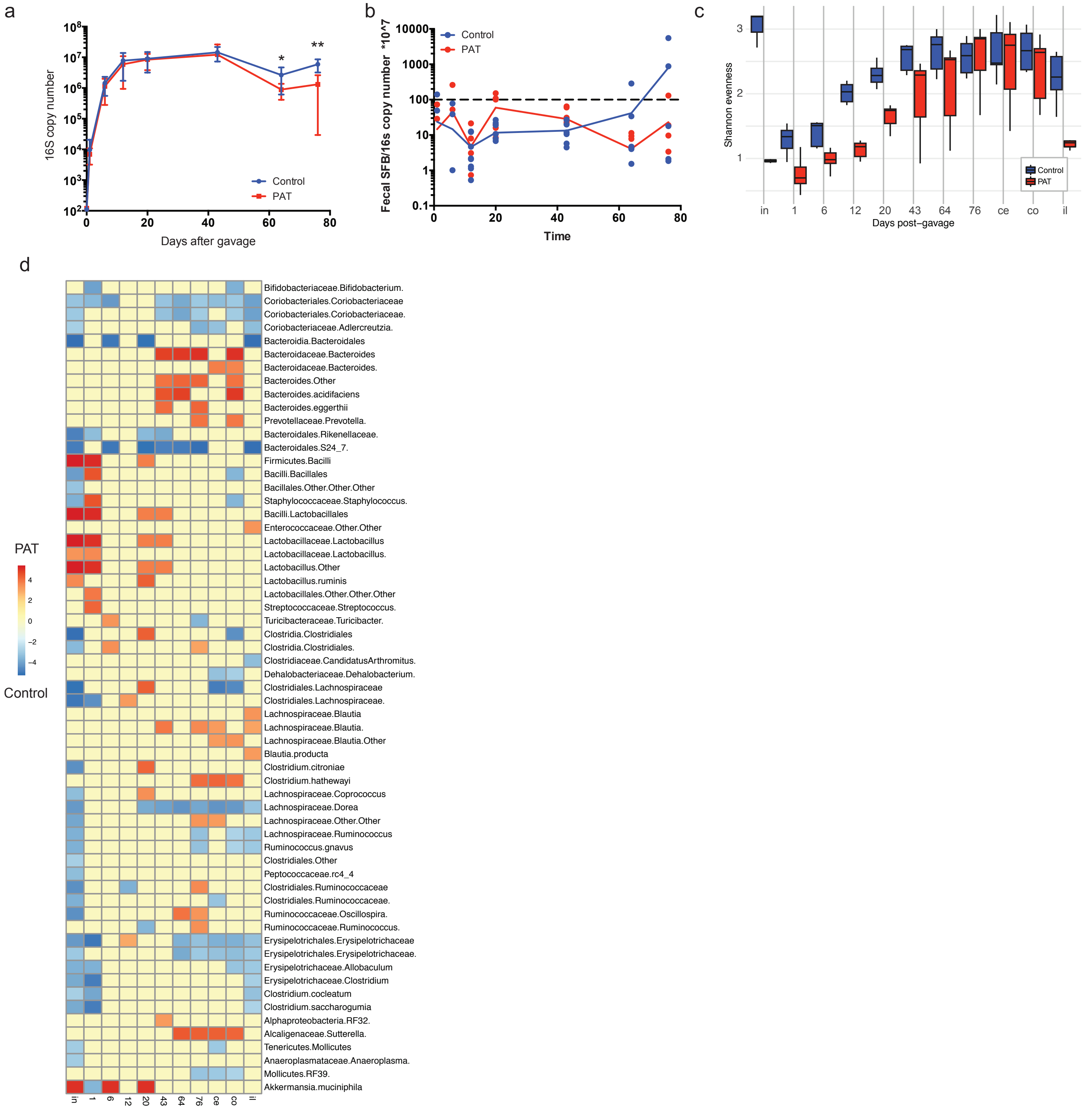

Supplementary Figure 2. Microbial community dynamics after conventionalization of germ-free mice with PAT-perturbed or control microbiota. (a) Mean (± SD) total 16S rRNA copy number over the course of the experiment between conventionalized Control (n=6) and PAT-perturbed (n=7) microbiota recipient groups (*p<0.05, **p<0.01). (b) Absolute SFB copy numbers normalized by total 16S rRNA in conventionalized groups (Control, n=6; PAT, n=7), lines represent group mean. Significance testing for (a) and (b) performed using the Mann-Whitney non-parametric test. (c) Median (± IQR) microbiota alpha diversity using the shannon evenness metric over time in germ-free mice conventionalized with control (n=6) or PAT microbiota (n=7), unrarified. Statistical analysis performed using two-sample t-test with Monte Carlo permutations, for statistical significance, see Supplementary Table 1. (d) Heatmap of significantly abundant taxa in inoculum (in), fecal, cecum (ce), colon (co), and ileum (il) of mice conventionalized with control or PAT microbiota over time (p<0.05) using the Linear discriminant analysis Effect Size (LefSe) statistical tool. Colors represent taxa significantly over-represented in control (blue) or PAT-perturbed (red) microbiota recipient mice; using the aheatmap R package.

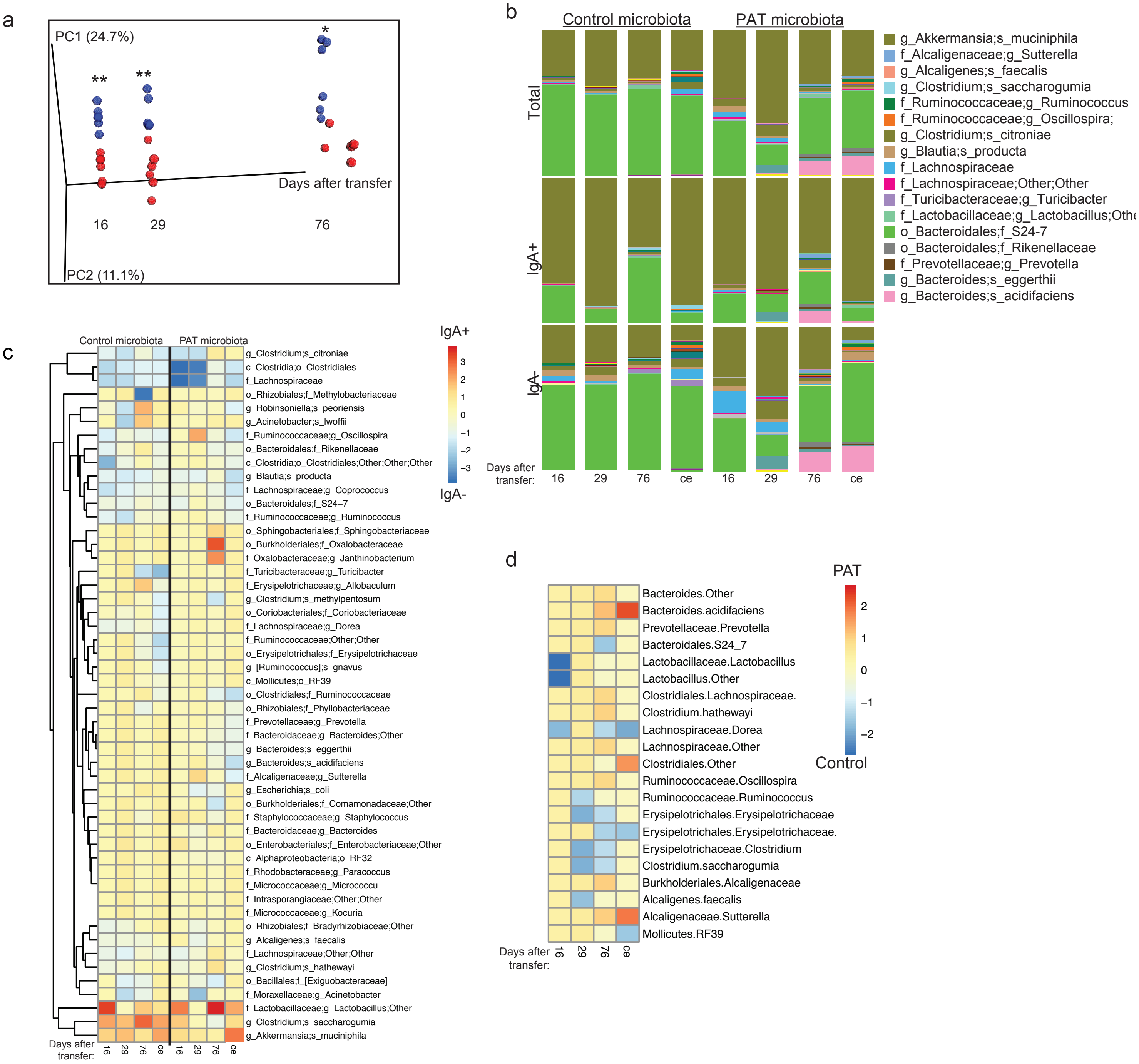

Supplementary Figure 3. Identification of IgA-bound taxa after conventionalization of germ-free mice with microbiota from PAT-exposed or control mice. (a) Unweighted UniFrac distances of IgA-bound bacteria shown as principal coordinates analysis (PCoA) by day after transfer of cecal contents from control (blue circles, n=6) or PAT-exposed mice (red circles, n=7). Statistical analysis was performed using the Adonis test, **p=0.003, *p=0.017. (b) Mean relative taxonomic abundances in fecal and cecal (ce) samples of total, IgA+ and IgA- fractions in recipient mice after conventionalization with PAT or Control microbiota. (c) Heat map depicting ICI scores in mice after conventionalization with Control or PAT microbiota over time in fecal and cecal (ce) samples. Colors represent taxa over-represented in IgA+ fractions (red) or IgA- fractions (blue). (d) Heatmap showing significantly abundant taxa (p<0.05) in the IgA+ fractions of mice conventionalized with control or PAT microbiota over time using the Linear discriminant analysis Effect Size (LefSe) statistical tool. Colors represent taxa over-represented in the Control (blue) or PAT-perturbed (red) microbiota recipient mice.

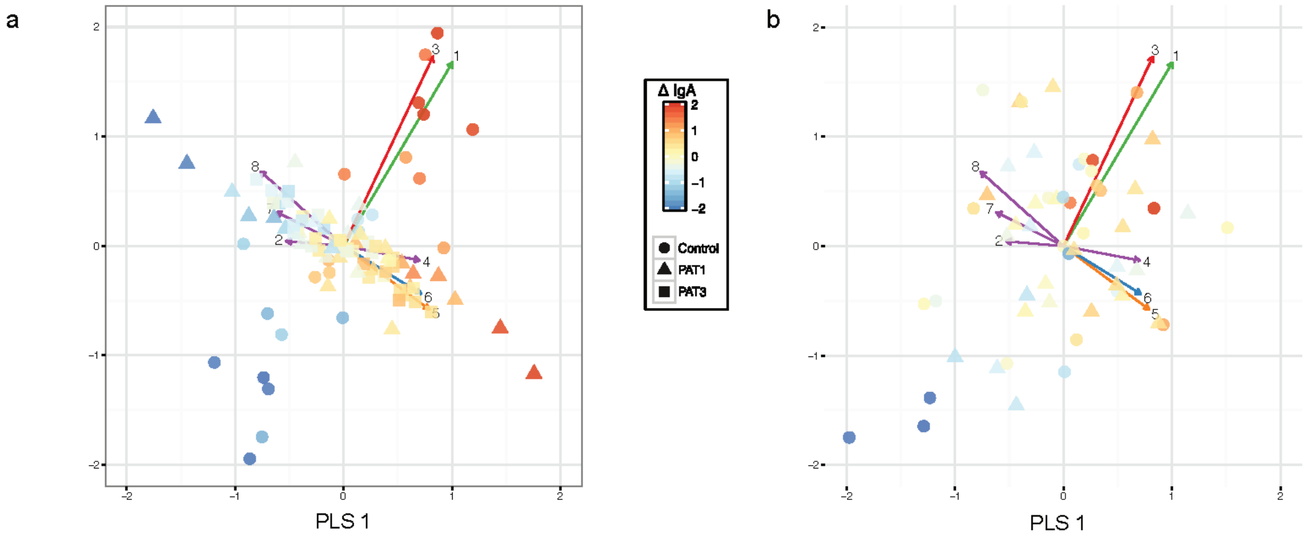

c

	Phylum	Class	Order	Family	Genus	Species	P-value	IgA
8	Firmicutes	Clostridia	Clostridiales	Lachnospiraceae			1.3e-03	−
2	Firmicutes	Clostridia	Clostridiales	Lachnospiraceae			< 1.0e-04	−
7	Firmicutes	Clostridia	Clostridiales	Lachnospiraceae	Clostridium	hathewayi	< 1.0e-04	−
5	Bacteroidetes	Bacteroidia	Bacteroidales	Rikenellaceae			3.5e-02	+
6	Proteobacteria	Gammaproteobacteria	Enterobacteriales	Enterobacteriaceae			3.3e-03	+
4	Firmicutes	Clostridia	Clostridiales	Lachnospiraceae	Blautia		< 1.0e-04	+
3	Actinobacteria	Actinobacteria	Bifidobacteriales	Bifidobacteriaceae	Bifidobacterium		< 1.0e-04	+
1	Firmicutes	Erysipelotrichi	Erysipelotrichales	Erysipelotrichaceae	Clostridium		< 1.0e-04	+

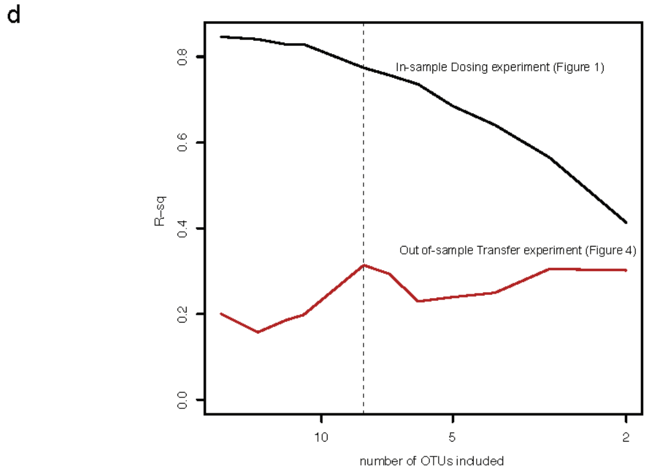

Supplementary Figure 4. Fecal microbial compositions are associated with changes in IgA. (a) Within-subject variances of center log ratio (clr)-transformed OTU compositions from the fecal microbiota experiment 1 (antibiotic dosing experiment) (See Figure 1), compositions are projected into a two-component subspace that maximally co-varies with within-subject IgA measurements (modeled by sparse partial least squares (sPLS) regression, r2=0.77). Results shown for fecal samples in 48 mice at days 27 and 43 (96 samples in total), in the dosing experiment. For the transfer experiment samples (See Figure 4), mice were sampled at days 6, 12, 20, 43, and 57 after transfer. Individual samples are each shown by an individual shape and the color scale represents natural log (ln) differences in intestinal sIgA levels from P27 to P40. In the biplot, lines are loading vectors and numbered by the corresponding taxa (in panel c) and colored by taxa family. (b) Model sparsity is determined by maximizing out-of-sample predictive power of the corresponding OTUs in the transfer experiment (r2=0.31). (c) Lineages of the eight selected OTUs, from dosing experiment (Figure 1), together with empirical p-values, computed using 2000 bootstraps, and the direction of association with IgA levels. (d) The sum of squared residuals (R-sq) vs. number of OTUs included in the multi-level PLS model. R-sq shows goodness of fit of in-sample prediction from fitted data from the dosing experiment and out-of-sample predictions for the transfer experiment over rebuilt models with the included OTUs. The final model was selected by maximizing the out-of-sample predictions (vertical dotted line).

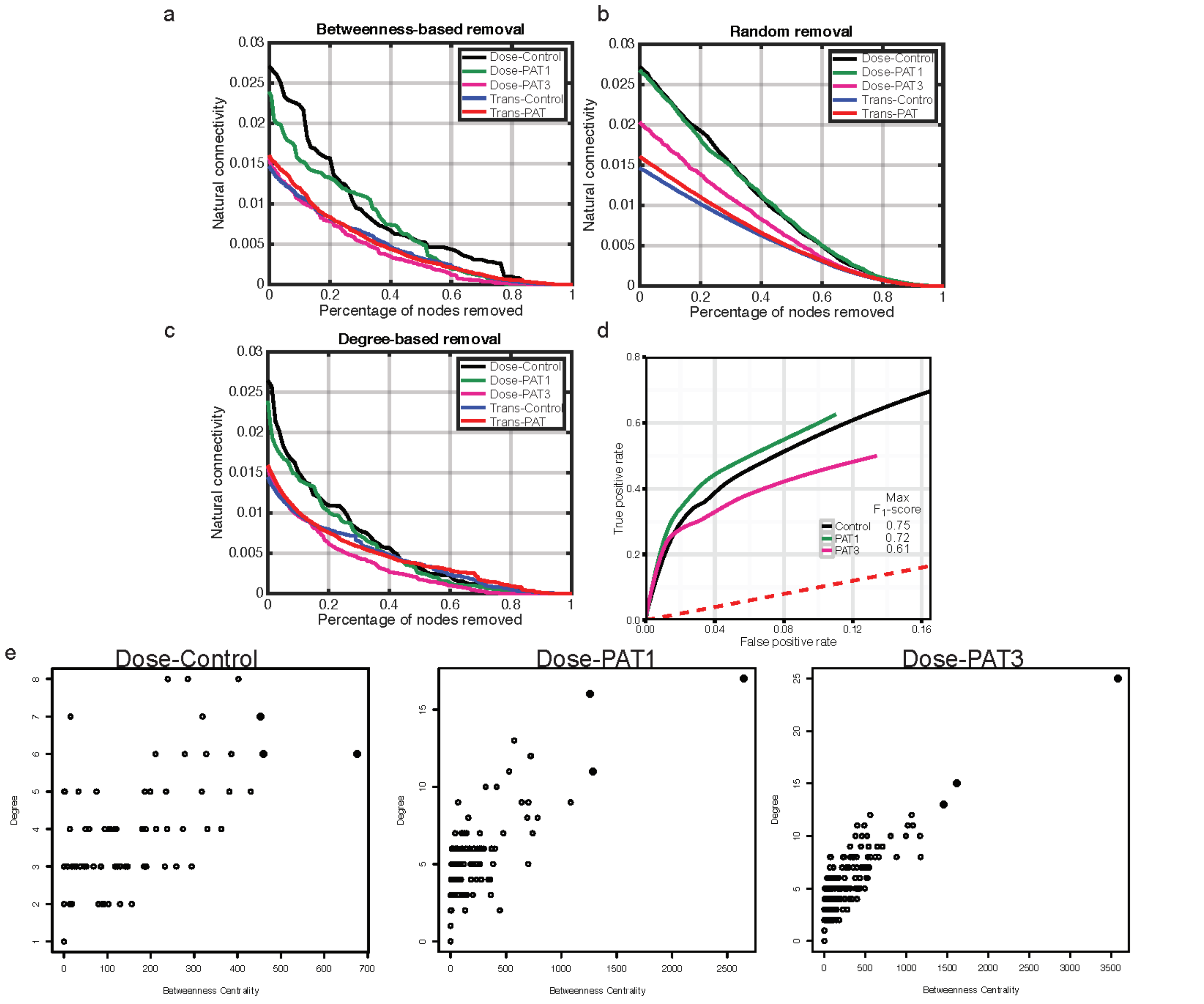

	Phylum	Class	Order	Family	Genus	Species
1	Proteobacteria	Gammaproteobacteria	Enterobacteriales	Enterobacteriaceae		
2	Firmicutes	Clostridia	Clostridiales	Lachnospiraceae	Blautia	producta
3	Firmicutes	Clostridia	Clostridiales	Lachnospiraceae		

	Phylum	Class	Order	Family	Genus	Species
1	Bacteroidetes	Bacteroidia	Bacteroidales	S24-7		
2	Bacteroidetes	Bacteroidia	Bacteroidales	S24-7		
3	Bacteroidetes	Bacteroidia	Bacteroidales	S24-7		

	Phylum	Class	Order	Family	Genus	Species
1	Firmicutes	Clostridia	Clostridiales	Ruminococcaceae	Oscillospira	
2	Firmicutes	Clostridia	Clostridiales	Lachnospiraceae		
3	Firmicutes	Clostridia	Clostridiales	Ruminococcaceae	Oscillospira	

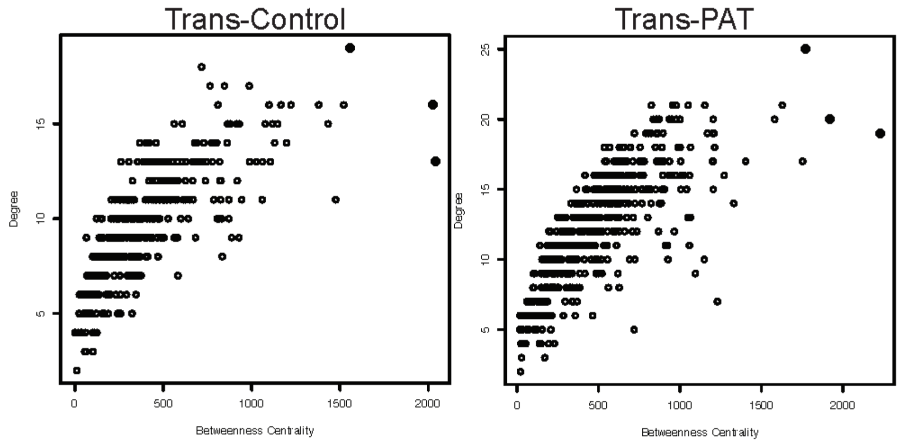

	Phylum	Class	Order	Family	Genus	Species
1	Proteobacteria	Gammaproteobacteria	Enterobacteriales	Enterobacteriaceae		
2	Firmicutes	Clostridia	Clostridiales	Lachnospiraceae		
3	Firmicutes	Clostridia	Clostridiales	Lachnospiraceae	Blautia	producta

	Phylum	Class	Order	Family	Genus	Species
1	Bacteroidetes	Bacteroidia	Bacteroidales	S24-7		
2						
3	Bacteroidetes	Bacteroidia	Bacteroidales	S24-7		

Supplementary Figure 5. PAT-induced alterations of network structures. (a-c) Natural connectivity was used to assess the robustness of microbial ecological interaction networks to sequential node removals from the dosing experiment (Dose) (Figure 1) and the transfer experiment (Trans) (Figure 4). The node removals were ordered by betweenness (a), random (b), or degree-based centrality (c). We repeated the random node removal experiment n=30 times and report the mean (standard error was within the thickness of the lines and hence not shown). Natural connectivity is shown as a function of the relative size of the network. (d) Recovery of edges from the transfer experiment-inferred networks to dosing experiment ('gold standard') networks was assessed by comparing Trans-Control/Dose-Control, Trans-PAT/Dose-PAT1 and Trans-PAT/Dose-PAT3 networks. The receiver operator characteristic (ROC) curves, assessing true vs false positive rates of prediction, and the maximum F1 score over the available edges, are shown. (e) For each OTU in the network, we computed degree and betweenness centrality (hollow circles). The highest ranked OTUs by both measures (filled circles) are interpreted to be keystone species, with the taxonomic lineage presented in tables below each panel.

Supplementary Table 1. Significance testing of Phylogenetic Diversity and Shannon evenness in Control, PAT1, and PAT3 groups over time or after conventionalization with control or PAT microbiota, related to Figures 1 and 4

p-values from Wilcoxon U testing[a] by day of life of pups (in Pups)

Group Comparison	Diversity metric	21	22	30	34	39	43	52
Control vs PAT1	**PD**	<0.001	<0.001	<0.001	<0.001	<0.001	<0.001	<0.001
	**Shannon**	<0.001	<0.001	<0.001	<0.001	<0.001	<0.001	<0.001
Control vs PAT3	**PD**	<0.001	<0.001	<0.001	<0.001	<0.001	<0.001	<0.001
	**Shannon**	<0.001	<0.001	<0.001	<0.001	<0.001	<0.001	<0.001
PAT1 vs PAT3	**PD**	0.890	0.122	<0.001	<0.001	<0.001	<0.001	0.017
	**Shannon**	0.737	1.000	0.263	<0.001	<0.001	<0.001	1.000

p- values from Wilcoxon U Testing by day of life of pups (in Dams)

Group Comparison	Diversity metric	5	10	12	30	34	39	43	Ce	Ile
Control vs PAT	**PD**	0.618	0.017	0.002	0.014	0.023	0.064	0.145	0.025	0.067
	**Shannon**	0.215	0.031	0.006	0.006	0.016	0.064	0.010	0.007	0.037

p-values from Wilcoxon U testing[a] by day after conventionalization (in transplant recipients)

Group Comparison	Diversity metric	Inoc	1	6	12	20	43	64	76	Ile	Ce	Co
Control vs PAT	**PD**	0.002	0.018	0.044	0.144	0.114	0.033	0.128	0.423	0.006	0.504	0.052
Control vs PAT	**Shannon**	0.001	0.006	0.037	0.006	0.006	0.082	0.125	0.980	0.006	0.569	0.240

Supplementary Table 2. Summary of Adonis and Anosim testing of Unweighted UniFrac distances, related to Figure 1

p-values from Adonis testing[a] by day of life of pups (in Pups)

Group Comparison	21	22	30	34	39	43	52
Control vs PAT1	0.001	0.001	0.001	0.001	0.001	0.001	0.001
Control vs PAT3	0.001	0.001	0.001	0.001	0.001	0.001	0.001
PAT1 vs PAT3	0.051	0.002	0.001	0.001	0.001	0.001	0.001

p-values from Anosim testing[a] by day of life of pups

Group Comparison	21	22	30	34	39	43	52
Control vs PAT1	0.001	0.001	0.001	0.001	0.001	0.001	0.001
Control vs PAT3	0.001	0.001	0.001	0.001	0.001	0.001	0.001
PAT1 vs PAT3	0.060	0.024	0.001	0.001	0.001	0.001	0.001

p-values from Adonis testing[a] by day of life of pups (in Dams)

Group Comparison	5	10	22	34	39	43	61
Control vs PAT	0.527	0.068	0.183	0.068	0.068	0.068	0.124

p-values from Anosim testing[a] by day of life

Group Comparison	5	10	22	34	39	43	61
Control vs PAT	0.503	0.436	0.436	0.436	0.436	0.436	0.436

[a]FDR-corrected

Supplementary Table 3. Fecal sIgA levels in Dams before PAT or Control exposure[a]

Time	sIgA levels/ Total protein x 10^3 Control (n=3)	PAT (n=7)	p-value
Breeding[b]	23.6 +/- 2.9	20.6 +/- 2.6	NS
Gestation day 16[b]	20.6 +/- 12.4	18.9 +/- 2.6	NS

[a] Mean ± SEM
[b] NS (not significant)

Supplementary Table 4. Ingenuity pathway analysis differentiating PAT and Control in pups and dams, showing the 12 most significant pathways for each

Pups

Pathway	Number of genes Total	Significant	% decreased	$-\log_{10}$ (p-value)
Antigen Presentation Pathway	18	12	100	9.0
Protein Ubiquitination Pathway	146	34	100	7.8
Superpathway of Melatonin Degradation	36	11	9	4.0
tRNA Charging	16	7	100	3.8
B Cell Development	12	6	100	3.7
Type I Diabetes Mellitus Signaling	62	14	100	3.4
Serotonin Degradation	37	10	20	3.2
OX40 Signaling Pathway	20	7	100	3.1
Graft-versus-Host Disease Signaling	21	7	100	3.0
Melatonin Degradation I	33	9	0	3.0
Nicotine Degradation II	33	9	0	3.0
T Helper Cell Differentiation	41	10	100	2.8

Dams

Pathway	Number of genes Total	Significant	% decreased	$-\log_{10}$ (p-value)
Putrescine Degradation III	12	2	0	2.75
Tryptophan Degradation X (Mammalian, via Tryptamine)	15	2	0	2.55
Dopamine Degradation	16	2	0	2.50
Noradrenaline and Adrenaline Degradation	19	2	0	2.35
Sorbitol Degradation I	1	1	0	2.27
Thyroid Hormone Biosynthesis	2	1	0	1.97
Heparan Sulfate Biosynthesis	32	2	50	1.91
Superpathway of Melatonin Degradation	36	2	0	1.81
Melatonin Degradation II	3	1	0	1.80
Serotonin Degradation	37	2	0	1.78
Glycerol Degradation I	4	1	0	1.67
LPS/IL-1 Mediated Inhibition of RXR Function	131	3	1	1.40
